# Thermophilic Fungi to Dominate Aflatoxigenic/Mycotoxigenic Fungi on Food under Global Warming

**DOI:** 10.3390/ijerph14020199

**Published:** 2017-02-17

**Authors:** Robert Russell M. Paterson, Nelson Lima

**Affiliations:** CEB—Centre of Biological Engineering, Campus de Gualtar, University of Minho, 4710-057 Braga, Portugal; nelson@ie.uminho.pt

**Keywords:** climate change, thermotolerant fungi, *Aspergillus flavus*, *Aspergillus fumigatus*, patulin, crops

## Abstract

Certain filamentous fungi produce mycotoxins that contaminate food. Mycotoxin contamination of crops is highly influenced by environmental conditions and is already affected by global warming, where there is a succession of mycotoxigenic fungi towards those that have higher optimal growth temperatures. Aflatoxigenic fungi are at the highest limit of temperature although predicted increases in temperature are beyond that constraint. The present paper discusses what will succeed these fungi and represents the first such consideration. Aflatoxins are the most important mycotoxins and are common in tropical produce, much of which is exported to temperate regions. Hot countries may produce safer food under climate change because aflatoxigenic fungi will be inhibited. The same situation will occur in previously temperate regions where these fungi have recently appeared, although decades later. Existing thermotolerant and thermophilic fungi (TTF) will dominate, in contrast to the conventional mycotoxigenic fungi adapting or mutating, as it will be quicker. TTF produce a range of secondary metabolites, or potential mycotoxins and patulin which may become a new threat. In addition, *Aspergillus fumigatus* will appear more frequently, a serious human pathogen, because it is (a) thermotolerant and (b) present on crops: hence this is an even greater problem. An incubation temperature of 41 °C needs employing forthwith to detect TTF. Finally, TTF in crops requires study because of the potential for diseases in humans and animals under climate change.

## 1. Introduction

Global warming due to climate change is becoming more certain and accepted. It is likely being exacerbated by human industrial activities, which at least offers the hope that it can be reduced by humans, in contrast to natural phenomena. The recent Paris agreement on climate change restricts increases to a maximum of 2 °C, and rapid, significant action is anticipated by many. Nevertheless, the consequences of global warming are becoming more evident with each passing year. Increases in air and ocean temperatures, rising sea levels and large-scale melting of ice and snow, since the beginning of the 20th century, represent signs of climate change [1]. Land-ocean temperature was ca. 0.8 °C warmer in 2000–2010 than at the beginning of the 20th century, and two thirds of the warming occurred since 1975 [2]. Global climate models give a more than 90% probability that the hottest seasons on record will represent the norm in many temperate locations.

Importantly, the crop growing season temperatures in the tropics and subtropics will exceed the most extreme seasonal temperatures recorded in the 20th century [1]. The worst consequences of climate change may not be felt until 2050 [3,4], but significant adverse effects are expected in the short term from more frequent extreme conditions. Current emissions will continue their impact in the future [3], and many hot countries will experience more extreme temperatures (e.g., Pakistan [4] and India [5]. Consequently, crops will be grown in areas with higher, and potentially more stressful, land surface and atmospheric temperatures. Crops may not adapt to increased temperatures, although maize is expected to acclimatise [6], where regions will become unsuitable, remain suitable or become suitable [7]. Drought stress is beneficial to opportunistic fungal pathogens that may not otherwise have an impact on crop hosts. Many of the mycotoxigenic micro-fungi are, at best, weakly pathogenic and they take advantage of host stress weakening resistance. Significant climate changes (e.g., increased temperatures and decreased water availability) provide an advantage to these pathogens.

Temperature increases due to climate change will increase the incidence and severity of crop pests and diseases [8]. Furthermore, developing effective plant disease management strategies in food crops requires the prediction of climate change impacts. Accelerated evolution and changing geographic distribution of plant diseases are the effects where forecasting systems are needed [9,10]. Accurate predictions are impossible if the wrong disease organisms are considered because they are superseded by novel pests [11] and climate is a potent selective force in populations that may alter host-parasite interactions [12]. Overall, climate change affects the status quo in three important crop areas: environment, plant host and pathogen which comprise the structure of a plant disease triangle. Many plant diseases involve a fungal pathogen, and many of these also have the potential to produce toxic secondary metabolites known as mycotoxins.

A major constraint to crop production is mycotoxins produced by micro-fungi contaminating crops [13,14]. Mycotoxins are in some cases very toxic [15,16] with only some fungi being responsible for mycotoxin production on specific crops [7,17,18]. Mycotoxins are associated with human and domesticated animal diseases, causing death in rare cases. Approximately seven mycotoxins (e.g., aflatoxin B1, aflatoxin G1, ochratoxin A, fumonisins, deoxynivalenol, patulin and nivalenol) have statuary limits in crops leading to rejection if the concentrations are high. For the purpose of this article, they are referred to as “conventional mycotoxins”. Mycotoxin production and fungal growth are affected highly by environmental conditions such as temperature and moisture [15,16] and much work has determined the effects on the growth and production of fungi currently affecting crops. In addition, many fungi found on food produce secondary metabolites, which are not considered formally as mycotoxins and are referred to herein as “potential mycotoxins”. The effects of climate change are considered in this current paper.

## 2. Effect of Climate Change on Mycotoxigenic Fungi

Reducing contamination by mycotoxigenic fungi is integral to food security [9] where existing mycotoxigenic fungi may become extinct, mutated or attenuated [11]. Some basic premises are: (a) crops planted in novel regions may have few mycotoxigenic fungi; (b) there may be a progression to higher temperature mycotoxigenic fungi (e.g., *A. flavus*) in currently temperate regions; (c) there will be some regions where crops will no longer grow, and so, there will be no mycotoxin problem [15,16]; and (d) there may be temperatures of 41 °C and above where aflatoxigenic fungi are out-competed by thermotolerant and thermophilic fungi (TTF).

Warm temperatures and high humidity are key factors for mycotoxigenic fungi [19] and influence their germination, growth, sporulation and mycotoxin production [20]. Not only will temperature increases alter growth and toxigenic potential of fungal pathogens, but also their ability to compete will be affected. Novel mycotoxin-commodity combinations are of further concern and allow new fungal genotypes with high aggressiveness and increased mycotoxin production [21], including TTF that are capable of producing novel secondary metabolites. Indeed, virulent fungi are displacing conventional ones at an increasing rate [22].

Extensive research to control contamination by mycotoxigenic fungi is ongoing [15]. Researchers have considered the effect of climate change on the physiology of conventional mycotoxin production [7,16,17,23] without considering in detail novel mycotoxigenic fungi and potential mycotoxins relevant to the future. Paterson and Lima [16] suggested *A. flavus* and *A. parasiticus* may become extinct in currently hot regions, a potential benefit to crop production. Furthermore, currently temperate regions may become inhibitory for the growth of aflatoxigenic fungi, albeit later than in hot countries. Overall, there will be regions where it is too hot for the common mycotoxin fungi to produce mycotoxins [11,16], sooner (e.g., 20 years) or later (e.g., 70 years). Adaptation of mycotoxigenic species will be important in 20 years [3,4] and a form of adaption by mutation is discussed in references [11,12,19], where advantageous sexual recombination and survival of resistant sclerotia may be possible. In the current authors’ view, more likely would be a succession of fungal species through the optimal growth temperature ranges which would occur quicker [11], and more evidence for this is provided in the Succession section below. Some species may have adapted from other species, such as within the fusaria where species complexes can occur. For example, the more toxigenic *F. graminearum* strain discussed in [24] and the Northland *Fusarium* population [25] may be adapted taxa. Forecasting of mycotoxigenic fungi and mycotoxin contamination in the future becomes an important area for research.

High temperature (but not above 41 °C) and drought are key for *A. flavus* growth and aflatoxin production. *A. flavus* and *A. parasiticus* are xerophilic fungi, which thrive under lower rainfall (and higher temperatures) [26]. Some studies of current mycotoxigenic fungi and conventional mycotoxins consider predominantly the effect of temperature and a_w_ and a few include CO_2_ levels [3,4]. However, there is no information on what will occur if these fungi cannot grow because of climate change [16]. Indeed, it is reported that temperatures reached 55.7 °C in Pakistan [4], which is inhibitory to the growth of *A. flavus*.

Many crops are produced in industrially undeveloped countries in hot climates where the lowest mycotoxin-containing commodities are exported to industrially-developed countries. Studying the effects of climate change on aflatoxigenic fungi may be irrelevant to these already hot countries, as they may become extinct [11,16]; an advantage for crop production in these countries. There are no known conventional mycotoxigenic fungi that can displace these fungi.

## 3. Effect of Climate Change on Mycotoxins

A key aspect of food and feed safety is the progression of conventional mycotoxin contamination in cereals due to climate change [26,27]. Food and feed contaminated with mycotoxins are most frequently found in warmer agricultural regions, and climate change is expected to negatively-affect quality [28]. More relevance is obtained if future potential mycotoxins are considered [11]. In general, changes in mycotoxins in crops will reflect changes in the producing fungi [29]. Different mycotoxins will dominate because the normal producing fungi have been outcompeted, and current research may not inform about future mycotoxin problems; therefore, we cannot rely on existing paradigms [11].

Aflatoxin outbreaks are most severe in tropical and subtropical areas around the world [21]: Aflatoxin production was optimum at 28–30 °C, but ceased at 37 °C [30], which are within the tropical temperature range. Low rainfall and maximum temperature affected aflatoxin contamination in maize in Georgia, USA [31]. Previously, in Europe, aflatoxin contamination was confined to imported foods, such as peanut cake, palm kernel, copra, and corn gluten meal [32]. These commodities from the tropics may be susceptible to secondary metabolites from TTF.

Postharvest, the fungi in grain silos can multiply more rapidly under warmer temperatures producing higher amounts of metabolic water, initiating spoilage and increasing contamination with mycotoxins. This means that the distribution and types of mycotoxins may change significantly, giving rise to new emerging mycotoxins [33]. What evidence is there of successions of mycotoxigenic fungi because of climate change?

## 4. Succession Events Involving Mycotoxigenic Fungi

Aflatoxigenic species from the tropics and subtropics may invade southern Europe, which increasingly is becoming more tropical. Hot and dry weather in Europe [16] contributed to a 2003 outbreak of *A. flavus* on crops in Italy, uncommon previously. *A. flavus* was able to colonize ripening maize by outcompeting the more common *Fusarium* species [15,34]. This caused an increase in aflatoxin B1 contamination in Europe, an interesting example of how mycotoxins can estimate the presence of producing fungi as a biochemical diagnostic method [29]. There were five notifications of aflatoxin M1 in milk in 2012, and all were related to an increase of aflatoxins in maize for animal feed from southeast Europe [1]. The maize survived a severe drought, making the crop vulnerable to infection by *A. flavus*/*A. parasiticus*, resulting in increased aflatoxins. A large survey [35] established aflatoxin contamination of corn, almonds and pistachios grown in southern Europe due to the recent development of a subtropical climate. Furthermore, a model developed for *A. flavus* growth and aflatoxin B1 production indicated that aflatoxin contamination will increase in maize from climate change. There was a clear increase in aflatoxin risk in areas in central/southern Spain, south Italy, Greece, north/southeast Portugal, Bulgaria, Albania, Cyprus and Turkey with +2 °C. That temperature increase is the limit specified by the recent Paris agreement mentioned earlier.

*A. flavus* was able to colonize maize at the ripening stage by outcompeting *Fusarium* species [36]. Drought and extreme elevated temperatures (>35 °C) resulted in a change from *Fusarium verticillioides* and contamination with fumonisins of maize, to *A. flavus* and aflatoxins [37]. Reduced sporulation occurred at dry conditions of ≤0.90 water activity (a_w_) [38], and *A. flavus* can even grow at 0.73 a_w_ and produce aflatoxins at 0.85 a_w_, while *F. verticillioides* growth is low at 0.90 a_w_ and fumonisins produced at >0.93 a_w_. A Serbian maize survey in 2009–2011, identified no aflatoxin contamination, although prolonged hot and dry weather in 2012 resulted in 69% of samples being contaminated with aflatoxins [39]. Increases in aflatoxins in maize may be from climate change in Hungary [40], and aflatoxin-producing *A. flavus* isolates in several maize fields were isolated in 2012–2013, whereas none were isolated 20 years before. Furthermore, mycotoxin levels in cereal and mixed feed samples collected in Hungary were examined [40], and aflatoxin B1 levels above the EU limit in 4.8% of the samples were observed. Aflatoxin in maize (2003) and milk (2007, 2011, 2012, 2013) originating from Hungary, Serbia, Romania and Slovenia has been detected within the framework of the Rapid Alert System for Food and Feed of the European Union [41]. Due to the extreme weather conditions in 2012 in Central Europe, aflatoxin contamination of maize and milk caused serious problems in Serbia, Romania and Croatia. Aflatoxins were also detected in maize kernels in Hungary after harvest in 2012 [42].

This succession phenomenon has also been observed, within the genus *Fusarium*, in other non-maize crops. *Fusarium graminearum* was the most abundant *Fusarium* on wheat in The Netherlands in the early 2000s, whereas *F. culmorum* was dominant in 1990s [43]. *F. graminearum* has increased on wheat in the UK, while *F. culmorum* is less copious [44]. In Germany, *F. culmorum* was prevalent, whereas *F. graminearum* dominated more recently [45]. *F. poae* dominated in Poland followed by *F. tricinctum*, *F. avenaceum*, *F. culmorum* and then *F. graminearum* [46] for which a significant increase in *F. graminearum* has now been observed [47]. T-2 and HT-2 toxin contamination are more prevalent on oats and barley in the UK, related to *F. langsethiae* detection in grains [44] and by implication, climate change. In addition, this species has become prevalent on barley in recent years in northern France [21] and produces toxins [36]. High temperatures favor growth of the fumonisin producer *Fusarium verticillioides* in maize [33]. The displacement of the formerly predominant species, *F. culmorum* and *Microdochium nivale*, by the more virulent plant pathogen *F. graminearum* as a result of warm European summers has been reported [33]. A more toxigenic 3-acetylated deoxynivalenol (3ADON) chemotype of *F. graminearum* replaced the 15-acetylated deoxynivalenol (15ADON) chemotype in Canada [47].

Novel strains that form unexpected toxins are also being discovered and may have resulted from changing environments. Such shifts in mycotoxigenic fungi may also lead to changes in the mycotoxin chemical profile. Minnesota, USA, for instance, has witnessed the emergence of a novel *Fusarium* isolate called the “Northland population”, which does not produce the trichothecenes deoxynivalenol or nivalenol [25].

## 5. Thermotolerant and Thermophilic Fungi

Climate change implies increases in temperature. Hence, more fungi that tolerate or prefer higher temperatures can be expected where crops can still be grown. Aflatoxigenic fungi would be readily outgrown by TTF (Table 1) if adapted to grow on the same crops. The original toxigenic fungi could (a) mutate [11]; (b) undergo horizontal gene and/or (c) favorable recombination events could happen over time to facilitate adaptation. However, it is likely that TTF would simply succeed the aflatoxigenic fungi as described in Section 4, because it would be quicker than the alternatives and many TTF produce secondary metabolites or mycotoxins, which could contaminate crops. The temperatures that favor TTF will be too high even for the most high temperature-aflatoxigenic fungi.

Stressed, modified and novel crops in hot climates will be threatened by infection from TTF, and a list of known potential mycotoxins and conventional mycotoxins from TTF are given in Table 1. It is interesting that patulin is the only conventional mycotoxins listed. Some TTF are present currently on crops, but less frequently isolated because they are outgrown by the mesophilic fungi at the normal incubation temperatures of 25 °C. The other mesophilic and non-mycotoxigenic fungi would also be inhibited by the higher temperatures.

TTF are of the microbiota that develop in heaped masses of self-heating plant material, piles of agricultural and forestry products and other accumulations of organic matter wherein the warm, humid and aerobic environment provides the conditions for development (Table 1). These include: composts, piles of hays, stored grains, wood chip piles, nesting material of birds and animals, animal dung, snuff, municipal refuse and other organic matter. Few studies have been conducted regarding the taxonomy and diversity of these fungi; however, they constitute heterogeneous genera [48]. Thermophily is restricted to species within *Thermomyces*, *Thermoascus* and *Rasamsonia* in the order Eurotiales. Furthermore, *Myceliophthora* comprises seven truly thermophilic species: *M. fergusii*, *M. fusca*, *M*. *guttulata*, *M. heterothallica*, *M. hinnulea*, *M. sulphurea* and *M. thermophila* [49]. Table 1 lists secondary metabolites and mycotoxins from some TTF [50]. However, if these particular fungi do not become established, then others adapted specifically to particular crops may do so.

The most dangerous member of those listed in Table 1 is *A. fumigatus*—a fungus often isolated from food which produces a range of potential mycotoxins. The fungus is the most serious human filamentous fungal pathogen [51], which is a more important threat than mycotoxin production. It is possible that farm workers, or those in contact with food, could be increasingly exposed to this fungus as climate change progresses: more exposure of immunocompromised or other at risk people could occur [52,53].

TTF could infect crops as they are adapted to similar substrates, and there would be little competition on the crops at the higher temperatures. A higher level of *A. fumigatus* in maize than *A. flavus* was found [54], indicating a wide occurrence under current conditions. The fungus was isolated from tobacco together with more recognized mycotoxin producing fungi [55], and *A. fumigatus* was frequently isolated from hazelnuts and walnut seeds [56], where incubation of cultures was at 45 °C. Gliotoxin is the most well-known of the metabolites produced by *A. fumigatus* and which could become present in crops. Table 1 lists the potential mycotoxins from *A. fumigatus* [50]. *Rhizomucor pusillus* was the other most common TTF in hazelnut and walnut seeds. *Humicola grisea* var. *thermoidae* and *Thermoascus aurantiacus* were isolated only rarely from walnut seeds and which are also thermophilic.

The potential mycotoxins and mycotoxins (in this case, only patulin (Table 1) from TTF may become important. Will current control/mitigation strategies control thermophilic fungi and minimize toxin contamination when marked global warming becomes normal [38]? Knowledge of environmental factors that may allow TTF to grow, survive, and interact with plants is important in order to better understand the variation in the population structures of these fungi, their interactions with crop plants, and the ability to produce mycotoxins [21], especially important under climate change.

To indicate how novel mycotoxins can become important, the European Commission has set recently maximum levels for citrinin in food supplements based on rice fermented with the red yeast *Monascus purpureus*, due to the nephrotoxicity of this mycotoxin [1], but which was not considered previously as an important mycotoxin. The European Food Safety Authority (EFSA) has delivered scientific opinions on the risks for animal and public health related to the presence of nivalenol, T-2 and HT-2 toxin, citrinin, beauvericin and enniatins in food and feeds; similarly for modified forms of the *Fusarium* toxins zearalenone, nivalenol, T-2, HT-2 toxins and fumonisins, many of which have received little recognition as mycotoxins [1].

Aflatoxin contamination is frequent in the tropics, but when the temperatures become too high aflatoxin contamination will decrease because *A. flavus* will not grow at the higher temperatures. This will be highly advantageous for the tropical countries in that their food will have low aflatoxin content. In this scenario, TTF may dominate, producing mycotoxins that will contaminate food, although they probably will be safer than aflatoxins.

## 6. Conclusions

An especially strict control system will require application to national agricultural commodities produced in currently temperate regions susceptible to contamination by aflatoxins. However, more attention will be paid in the future to the other regulated and unregulated mycotoxins [1], especially toxic secondary metabolites produced by TTF.

When isolating fungi from crops subsequently, the incubation temperatures in the laboratory may require increasing to above 41 °C, or more, to represent the new field conditions, and it may be necessary to consider TTF as novel risks. Specific methods for detecting multiple TTF metabolites may be required as an initial step to study their importance. *A. fumigatus* may pose the biggest threat from human infection apart from its ability to produce potential mycotoxins. Patulin may become increasingly important under climate change. TTF merit greater attention as threats to crops under climate change.

## Figures and Tables

**Table 1:** Thermotolerant and thermophilic fungi and associated secondary metabolites (potential mycotoxins) or conventional mycotoxins that could become important with respect to climate change [57,58,59]. Updated names are indicated in the table by reference [48].

Fungus	Synonyms	Temperature Opt °C	Temperature Max °C	Mycotoxin/Secondary Mycotoxins or Potential Mycotoxins	Comment
*Aspergillus flavus*		35	42	Aflatoxins	Common on many crops and foods
*A. fumigatus*		37	65	Gliotoxin, fumigatins, fumigaclavines, fumiquinazolines, fumitremorgins, verruculogens, helvolic acids	
*Byssochlamys verrucosa*		20–53		Contains patulin gene but compound not detected [49]	
*B. nivea*			46 at least [60]	Patulin	Well known patulin producer
*Canariomyces thermophile*		45			
*Chaetomium mesopotamicum*		45	52		
*C. thermophile*	*C. thermophilum*, *C. thermophilium*	45–55	58–61		Decomposing wheat straw, mushroom compost, vegetable detritus
*Coonemeria aegyptiaca*	*Thermoascus aegyptiacus*, *Paecilomyces aegyptiaca*	40	55		
*Co. crustacea*	*Thermoascus crustaceus*, *Dactylomyces crustaceus*, *Paecilomyces crustaceus*	40	60		Bagasse
*Co. verrucosa*	*Thermoascus crustaceus*	30–40	55		
*Dactylomyces thermophilus*	*Thermoascus thermophilus*, *Thermoascus aurantiacus* (misapplied name) 40–45	40–45			Birds‘ nest, wood and bark of *Pinus*, plant debris
*Malbranchea cinnamomea*	*Trichothecium cinnamomeum*, *Thermoidium sulfureum*, *Malbranchea pulchella* var. *sulfurea*	45	57		Composting heaps, wheat straw compost, stacked tobacco leaves, peanut kernels, hen-house litter, silage
*Melanocarpus albomyces*	*Myriococcum albomyces*, *Thielavia albomyces*	45	57		Mushroom compost. decomposing wheat straw, grass compost.
*M. thermophiles*	*Thielavia minuta* var. *thermophila*	35	50		
*Myceliophthora fergusii* [48]	*Thielavia thermophila*, *Myceliophthora fergusii*, *Chrysosporium fergusii*	50	60		Composts
*Myc. Hinnulea*		40–45	50		
*Myc. thermophila*	*Sporotrichum thermophilum/thermophile*, *Chrysosporium thermophilum*, *Myceliophthora indica*, *Corynascus heterothallicus*	45–50	55	Estatin A and B	Wood pulp
*Myriococcum thermophilum*		45	53		Horse manure-wheat straw compost
*Paecilomyces saturatus*		50	55	Patulin	This species forms a component of the *P. variotii* complex [61]. Ubiquitous contaminants of foods and raw materials.
*Rasamsonia byssochlamydioides* [48]	*Paecilomyces byssochlamydioides*	40–45	50		
*R. emersonii* [48]	*Geosmithia emersonii*; *Talaromyces duponti* and *Penicillium duponti (misapplied names)*	40–45	55		Compost, piles of wood chips, peat, sugarcane bagasse, palm oil kernels,
*Rhizomucor miehei*	*Mucor miehei*	35–45	57		Hay, stored barley, compost.
*Rh. pusillus*	*Mucor pusillus*	35–45	55		Mainly on composting and fermenting substrates like compost, wheat straw, hay, seeds of cacao, barley, oat, maize and wheat, groundnuts, pecans,
*Scytalidium thermophilum*	*Torula thermophila*, *Humicola grisea* var. *thermoidea*, *Humicola insolens*	40	58		Mushroom compost, wood chips.
*Stilbella thermophila*		35–50	55		Mushroom compost,
*Talaromyces duponti* [48]	*Penicillium duponti*	45–50	60	1. Talathermophilins: 2. Thermolides	Guayule shrub, fermented straw, compost,
*Thermoascus aurantiacus*		49–52	61	Contains patulin gene but compound not detected in two growth media [49].	Agricultural products, including maize stored in sub-Sahara Africa and olive and olive cake in Morocco and in food-related environments spoilage in various processed tea and fruit juice. Heated hay, peat, cacao husks, mushroom compost, stored grains, self-heated wood chips, chaff, tobacco, sawdust.
*Thermomyces ibadanensis*		42–47	61		Oil palm kernel stacks Compost
*Thermomyces lanuginosus*	*Humicola lanuginosa*	45–50	60	Thermolides, bacterial-like hybrid macrolactones	Compost. moist oats, cereal grains, mushroom compost, hay, leaf mold peat, garden compost, various plant substances.
*Thermomyces stellatus*	*Humicola stellata*	40	50		Moldy hay and soil
*Thielavia australiensis*		35–40	50		
*Thielavia pingtungia*		40	50		Sugarcane field
*Thielavia terrestris*	*Allescheria terrestris*, *Acremonium alabamensis (anamorph)*	40–45	52		Needles of *Pïnus taeda*.
